# Tackling Loneliness Among Patients With Borderline Personality Disorder in the Arab World: A Sociocultural Perspective

**DOI:** 10.7759/cureus.76346

**Published:** 2024-12-24

**Authors:** Syed Ali Bokhari, Beenish Mujahid, Bashayer M Almaazmi

**Affiliations:** 1 Psychiatry, Al Amal Psychiatric Hospital, Emirates Health Services, Dubai, ARE

**Keywords:** arab world, borderline personality disorder (bpd), cultural sensitivity, loneliness, personality disorders, psychiatry, stigma

## Abstract

Loneliness, a complex and multifaceted global issue, often affects individuals with borderline personality disorder (BPD), characterized by unstable relationships, poor self-image, and impulsive behavior. This paper explores the experience of loneliness among Arab patients with BPD, highlighting sociocultural challenges and barriers to seeking help. Cultural stigma, often tied to religious beliefs, significantly impedes mental healthcare in Arab societies. BPD is frequently misunderstood as divine punishment, leading individuals to seek faith healers instead of professional help. High expressed emotion (EE) in caregivers, stigma from healthcare providers, and disruptions due to wars further exacerbate isolation and loneliness among patients with BPD. Addressing BPD in the Arab world requires culturally and religiously informed approaches. Public awareness campaigns and collaboration with media and local authorities are essential to destigmatize BPD. Integrating traditional healing practices with therapy, tailored family interventions, and culturally adapted dialectical behavior therapy (DBT) can improve treatment outcomes. Islamic-based psychotherapy (IBP) offers a promising approach, combining social connection and evidence-based techniques. This multifaceted approach can create a supportive environment, ultimately reducing loneliness.

## Editorial

Introduction

Loneliness is an expanding worldwide phenomenon, complex and multifaceted. The definition of the term is subjective in nature; however, it is highlighted by an absence of social contact and belongingness, causing emotional suffering due to social isolation [1]. Borderline personality disorder (BPD) is a mental health condition highlighted by unstable interpersonal relationships, poor self-image, emotional lability, impulsive behavior with emphasis on suicidal attempts and self-harm, stress-related paranoid ideation, feelings of emptiness, and, at times, severe dissociation. As per the DSM-V, to receive the diagnosis, this must be present in multiple contexts and it must result in significant suffering or social impairment [2]. Bearing in mind the social and psychological toll the diagnosis can take on a person with BPD, it is inevitable, almost, that they experience isolation, disconnectedness, and, of course, loneliness [3].

Individuals with BPD often exhibit heightened sensitivity to rejection, intense fear of abandonment, and difficulties in regulating emotions. These characteristics can lead to unstable interpersonal relationships and behaviors that may be perceived as unpredictable or overly intense, potentially hindering social acceptance. For instance, the tendency to oscillate between idealizing and devaluing others, known as “splitting,” can create challenges in maintaining consistent and healthy social interactions [4].

This editorial aims to delve into the experience of loneliness among Arab patients diagnosed with BPD within the Arab region. It highlights the importance of understanding the sociocultural nuances that influence loneliness, the unique challenges faced by individuals with this diagnosis, and the barriers that hinder them from seeking help and support.

Challenges

Cultural stigma tied to religion significantly hinders mental health care in Arab societies. Personality disorders are frequently misinterpreted as divine retribution or attributed to supernatural phenomena such as jinn possession or the evil eye. These beliefs reflect a deeply rooted cultural heritage, encompassing traditional customs, practices, and collective wisdom passed down through generations, forming a vital connection to the community’s identity. However, such perceptions frequently lead individuals to prioritize seeking faith healers over professional mental health services, further limiting access to effective care. Fear of social isolation and the need to maintain social standing in tight-knit religious communities also contribute to avoiding professional mental health services. Additionally, a lack of mental health education (or “literacy”) exacerbates the problem [5].

High expressed emotion (EE) caregivers in Arab families face significant emotional distress and strain due to intense interactions with mentally ill relatives. This can lead to conflicts, chronic stress, anxiety, depression, and burnout. Cultural norms that emphasize familial responsibility increase feelings of guilt and self-blame among BPD patients, further isolating them [6].

Individuals with BPD face stigma from healthcare providers, leading to institutional isolation and loneliness. Inadequate training on BPD among healthcare providers results in misunderstandings and stigmatization. The lack of integrated care pathways complicates the situation, causing feelings of rejection and mistrust among BPD patients [7].

In Arab countries affected by wars and military conflicts, healthcare systems have been severely disrupted, leading to a scarcity of mental health resources. Displacement in these regions often results in social isolation and loneliness, as individuals are separated from their support networks. However, stigma, limited infrastructure, and a shortage of trained professionals are issues faced across the Arab region, further highlighting the urgent need for increased mental health resources [8]. Displacement often leads to social isolation and loneliness as individuals are separated from their support networks. These challenges are further compounded by the collectivist cultural values of Arab societies, which place significant emphasis on family honor, tradition, and interconnectedness. This orientation frequently fosters a culture of silence around sensitive issues such as mental health, in order to protect the family’s reputation. Within such societies, seeking mental health care may be perceived as bringing shame or dishonor to the family, prompting individuals to avoid professional help to maintain social standing [9]. The strong emphasis on family and community also heightens stigma, as behaviors deviating from social norms are often regarded as reflections on the entire family [10]. Limited infrastructure, a shortage of trained professionals, and these cultural dynamics further exacerbate the mental health crisis, highlighting the urgent need for increased resources [11]. Addressing these issues necessitates culturally informed care that respects family values while encouraging help-seeking behaviors, ensuring mental health professionals can navigate these sensitivities effectively.

Addressing BPD in the context of gender dysphoria is also crucial, given the widely varying prevalence rates, reported to range from 15% to 80% [12]. In the Arab region, the intersection of gender dysphoria and BPD significantly heightens feelings of loneliness, largely influenced by cultural sensitivities and rigid societal norms. Prevailing attitudes often lead to social ostracism and familial rejection, intensifying the internal conflict and emotional distress faced by individuals navigating both gender dysphoria and BPD.

Recommendations

Effectively addressing BPD in the Arab world countries requires a culturally and religiously sensitive approach, given the pervasive stigma that significantly impedes help-seeking behaviors [10,13]. Public awareness campaigns, in collaboration with media and local authorities, are crucial to destigmatize BPD and disseminate accurate information about available supports as well as address the current underdiagnosis of the condition and its societal impacts (e.g., domestic violence, substance abuse, and suicide) [14].

Integrating traditional healing practices into therapy can further reduce stigma and improve treatment outcomes by fostering trust and collaboration with faith healers, who hold significant influence in Arab communities [15,16]. Family therapy, tailored to respect familial structures while empowering patients, is pivotal in Arab culture [17]. Culturally adapted interventions, such as psychoeducation and skills training, can equip families with tools to navigate BPD challenges, promoting reconciliation within families [18-20].

Dialectical behavior therapy (DBT), the gold standard treatment for BPD, can be enhanced through cultural adaptation. Incorporating religious values like patience, reliance on God, self-compassion, and mindfulness into DBT techniques resonates with Arab patients [21,22]. Islamic-based psychotherapy (IBP) offers a promising avenue, integrating social connection and community building with evidence-based techniques to address loneliness in BPD, aiming to promote emotional regulation and a sense of belonging [23]. Shared decision-making with the patient is crucial for fostering trust, empowerment, and self-belief and developing coping mechanisms for identified triggers [24]. It also allows for the potential exploration of alternative treatments like functional analytic psychotherapy [25].

Conclusions

In the Arab region, cultural factors play a significant role in shaping the experience of loneliness among individuals with BPD. Tackling this issue requires a multifaceted approach that prioritizes reducing stigma. Public awareness campaigns and collaboration with faith healers can encourage help-seeking behaviors, while culturally sensitive therapeutic interventions that incorporate Islamic values can empower patients. Greater investment in research is needed to improve understanding of BPD and its co-occurring conditions, particularly in the Arab world. Furthermore, enhancing the training of healthcare professionals in BPD, alongside the adoption of culturally appropriate diagnostic tools for early identification, can substantially improve patient outcomes. By integrating these strategies, a more supportive environment can be fostered, ultimately reducing loneliness and improving the quality of life for Arab individuals with BPD.
